# *Cronobacter,* the emergent bacterial pathogen *Enterobacter sakazakii* comes of age; MLST and whole genome sequence analysis

**DOI:** 10.1186/1471-2164-15-1121

**Published:** 2014-12-16

**Authors:** Stephen J Forsythe, Benjamin Dickins, Keith A Jolley

**Affiliations:** School of Science and Technology, Nottingham Trent University, Clifton Lane, Nottingham, NG11 8NS UK; Department of Zoology, University of Oxford, Oxford, OX1 3PS UK

**Keywords:** Emergent bacterial pathogen, *Cronobacter*, MLST, Genomic analysis

## Abstract

**Background:**

Following the association of *Cronobacter* spp. to several publicized fatal outbreaks in neonatal intensive care units of meningitis and necrotising enterocolitis, the World Health Organization (WHO) in 2004 requested the establishment of a molecular typing scheme to enable the international control of the organism. This paper presents the application of Next Generation Sequencing (NGS) to *Cronobacter* which has led to the establishment of the *Cronobacter* PubMLST genome and sequence definition database (http://pubmlst.org/cronobacter/) containing over 1000 isolates with metadata along with the recognition of specific clonal lineages linked to neonatal meningitis and adult infections

**Results:**

Whole genome sequencing and multilocus sequence typing (MLST) has supports the formal recognition of the genus *Cronobacter* composed of seven species to replace the former single species *Enterobacter sakazakii*. Applying the 7-loci MLST scheme to 1007 strains revealed 298 definable sequence types, yet only *C. sakazakii* clonal complex 4 (CC4) was principally associated with neonatal meningitis. This clonal lineage has been confirmed using ribosomal-MLST (51-loci) and whole genome-MLST (1865 loci) to analyse 107 whole genomes via the *Cronobacter* PubMLST database. This database has enabled the retrospective analysis of historic cases and outbreaks following re-identification of those strains.

**Conclusions:**

The *Cronobacter* PubMLST database offers a central, open access, reliable sequence-based repository for researchers. It has the capacity to create new analysis schemes ‘on the fly’, and to integrate metadata (source, geographic distribution, clinical presentation). It is also expandable and adaptable to changes in taxonomy, and able to support the development of reliable detection methods of use to industry and regulatory authorities. Therefore it meets the WHO (2004) request for the establishment of a typing scheme for this emergent bacterial pathogen. Whole genome sequencing has additionally shown a range of potential virulence and environmental fitness traits which may account for the association of *C. sakazakii* CC4 pathogenicity, and propensity for neonatal CNS.

**Electronic supplementary material:**

The online version of this article (doi:10.1186/1471-2164-15-1121) contains supplementary material, which is available to authorized users.

## Background

### Next generation sequencing and multi-allelic profiling of emergent bacterial pathogens

Sequence-based methods for bacterial identification started with single locus sequencing, such as the 16S rRNA gene. This can differentiate isolates between phylum to genus level and often to species level, but no further [1]. The application of multilocus sequence typing (MLST), typically 7 loci, enables the recognition of bacterial sequence types (STs) and clonal complexes (CCs). These loci were initially sequenced individually with specifically designed primers. Nowadays, the application of Next Generation Sequencing (NGS) has facilitated whole genome sequencing for the equivalent cost of 7-loci and therefore has greatly increased the number of loci that can be used for strain discrimination and definition. The use of ribosomal MLST (rMLST) with 53 loci defined has already been used to define established bacterial pathogens to the strain level [2]. The use of whole genome MLST (>500 loci) to clone level is now possible and has been applied in epidemiological studies for pathogen typing, e.g. *Neisseria meningitidis*, methicillin-resistant *Staphylococcus aureus* and bacterial population genomics of *Campylobacter* [3–6].

### The emergent bacterial pathogen *Cronobacter*and the FAO-WHO call for molecular typing methods

Bacteria causing neonatal infections naturally attract attention and invoke a sense of injustice. Following the recognition of *E. sakazakii* (former name of *Cronobacter* genus) as the causative agent of fatal infections of neonates due to contaminated reconstituted infant formula [7–9] there were three FAO-WHO risk assessment expert group meetings [10–12] on the microbiological safety of these products on the international market. This was the first occasion that the WHO had aimed to control a foodborne bacterial pathogen. However at that time there had been few studies of relevance to help with imposing international control measures. Also the organism had undergone a number of taxonomic evaluations based on phenotypic tests resulting in confusing and conflicting information hampering the international control of this emergent bacterial pathogen by the development of specifc detection methods. Isolates had been variously described as *Praschechia flavescens* and ‘yellow-pigmented *Enterobacter cloacae’*, before a key study renaming them as *E. sakazakii* in 1980 and dividing the species into 15 biotypes [13]. Subsequently in order to reduce the risk of further neonatal infections, the FAO-WHO executive encouraged the setting up of appropriate detection and molecular typing schemes [10]. These are required for the adequate monitoring of sources and vehicles of the bacterium, in order to reduce the risk of neonatal exposure. These key points from the FAO-WHO executive summary are reproduced in Additional file 1: Box 1.

To date NGS of bacteria has primarily focused on well described organisms of clinical relevance such as *Escherichia coli* and *Salmonella*. This article, instead, considers the application of NGS to the newly emergent bacterial pathogen *Cronobacter* which was totally unlike *Salmonella* or *E. coli* with the lack of former information on its habitats, diversity, physiological or virulence traits The three tasks set by the FAO-WHO expert group (Additional file 1: Box 1) form the basis for this article to illustrate how using NGS has led to the establishment of the *Cronobacter* PubMLST genome database, and enabled the improved understanding and control for this emergent bacterial pathogen of international importance. Each task will be covered below as a discourse.

## Results and discussion

### Multilocus sequence typing of *Cronobacter*spp. and the *Cronobacter*PubMLST database

The need for an internationally validated detection and molecular typing method for the genus *Cronobacter* is needed given the severe outcomes of infections in neonates and the attributed link to contaminated powdered infant formula on the international market [10–12]. The *Cronobacter* PubMLST database includes the MLST profiling of isolates from all reported outbreaks around the world. Since the database also contains the metadata for over 1000 isolates an informed understanding of the diversity and sources of the organism can now be obtained. The MLST scheme covers all recognised species of *Cronobacter* genus, to better quantitate the intraspecific and interspecific diversity of the genus as well as potentially characterize the strains according to virulence groupings and source. It also enables the retrospective analysis of historic cases and outbreaks following re-identification of those strains which otherwise would have been lost due to taxonomic re-evaluations.

The organism initially was only known as a single species, *Enterobacter sakazakii*, composed of various biotypes based on phenotyping [13]. The application of 16S rRNA and *dnaG* gene sequencing led to the initial recognition that the organism was in fact composed of several species, and formed a unique genus that was distinguishable from *Enterobacter* [14]. The new genus was named *Cronobacter* to reflect the association of the bacterium with infant death (Kronos – Greek mythological god) [15].

**Figure 1:**
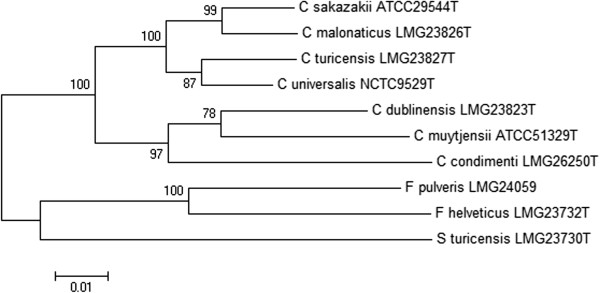
**Maximum likelihood tree of the seven multilocus sequence typing loci (3036 base pair concatenated length) for the members of** ***Cronobacter*** **genus and closely related** ***Franconibacter*** **and** ***Siccibacter*** **genera.** The tree was drawn using MEGA 6.05 (http://www.megasoftware.net/) with 1000 bootstrap replicates.

However 16S rRNA gene sequencing was unable to distinguish between all *Cronobacter* species and biotyping based on phenotypic tests gave subjective results. In order to overcome various limitations of phenotyping and 16S rDNA sequence analysis, the *Cronobacter* seven-loci MLST scheme was developed [16, 17]. The *Cronobacter* MLST scheme requires the partial sequence analysis of seven housekeeping genes: ATP synthase b chain (*atpD*), elongation factor G (*fusA*), glutaminyl tRNA synthetase (*glnS*), glutamate synthase large subunit (*gltB*), DNA gyrase subunit B (*gyrB*), translation initiation factor IF-2 (*infB*) and phosphoenolpyruvate synthase A (*ppsA*). When concatenated together these sequences provide 3036 nucleotides; the analysis of which is termed multilocus sequence analysis (MLSA). A MLSA phylogenetic tree of the *Cronobacter* genus and the closely related genera *Franconibacter* and *Siccibacter* is shown in Figure 1. A current description of the *Cronobacter* genus is given in Additional file 1: Box 2.

Before 2004 the number of *Enterobacteriaceae* (except for *Salmonella*) permitted in powdered infant formula was <100 cfu/g. These specifications had not been revised since 1979. However following the concern of the serious implications following *Cronobacter* infections of neonates, the microbiological criteria changed such that any member of the *Cronobacter* genus should not be detectable in 10 g test volume quantities of powdered infant formula [18]. Reliable detection of *Cronobacter* spp. has also been problematic due to the genus being poorly described. Prior to 2004, isolates were recovered using the general *Enterobacteriaceae* differential agar VRBGA, and then identified using a non-specific commercial phenotyping test kit resulting in considerable opportunity for false negative results [19]. While much of this topic is outside the scope of this article, it is pertinent to note that one biochemical trait that has predominated in the development of specific chromogenic agar is the constitutive expression of a maltose uptake mechanism and α –glucosidase [20]. This will be reconsidered in the last section with respect to taxonomy of the genus.

### Sources and vehicles of *Cronobacter*spp

Investigating 1007 isolate entries in the *Cronobacter* MLST database reveals for the first time the temporal, geographic and source diversity of the organism; Table 1. The earliest isolate (*C. sakazakii* NCIMB 8282) was from dried milk powder in 1950; the genome of which has now been published and is one of 107 genomes which can now be analysed via the PubMLST database [21]. *Cronobacter* strains have been isolated from 36 countries, and are from clinical (20.4%), infant formula (21.6%), food and food ingredients (14.2%), environmental (35.15%) and other sources such as water (4%); Tables 1 and 2. *Cronobacter* spp. isolation from plant material (wheat, rice, herbs, and spices) and various food products has previously been reported [22]. However, due to the history of concern, it is not surprising that a large portion of strains in the database are clinical or from powdered infant formula in origin.

**Table 1:** **Summary of** ***Cronobacter*** **isolates in the** ***Cronobacter*** **PubMLST database**

Species	Number of strains (%)	Number of STs ^a^	Number of genomes	Earliest isolate	Countries	Source				
						Clinical	Infant formula	Food and ingredients	Environment	Other
*C. sakazakii*	726 (72.1)	155	73	1950	33	19.8^b^	23.8	32.8	16.3	7.3
*C. malonaticus*	136 (13.5)	53	14	1973	17	36.0	16.2	36.8	7.4	3.7
*C. dublinensis*	59 (5.9)	44	9	1956	11	5.1	13.6	47.5	6.8	27.1
*C. turicensis*	41 (4.1)	26	6	1970	12	17.1	12.2	36.6	22.0	12.2
*C. muytjensii*	35 (3.5)	14	2	1988	10	2.9	28.6	45.7	2.9	20.0
*C. universalis*	9 (0.9)	5	2	1956	6	11.1	0.0	55.6	11.1	22.2
*C. condimenti*	1 (0.1)	1	1	2010	1	0	0	100	0	0
Total	1007	298	107		36	20.4^c^	21.6	14.2	35.1	8.7

^a^Sequence type.

^b^Percentage of species total.

^c^Percentage of genus total.

**Table 2:** **Profile of the source for the major sequence types in** ***C. sakazakii*** **and** ***C. malonaticus***

Cronobacter species	Clonal group	Number of profiled isolates	Percentage of species isolates	Infant (<1 y) ^b^				Child (>1 y)	Adult (>18 y)	Unknown ^e^	Source				
				Uncertain ^c^	Meningitis	NEC ^d^	Septicaemia				Clinical total	Infant formula	Food & ingredients	Environment	Others
C. sakazakii (726)^a^	CC4	195	26.9	4.6	10.0	5.1	4.1	2.1	16.9	2.0	45.1	24.6	8.2	16.9	5.6
	CC1	80	11.0	2.5	3.8	0.0	1.3	1.3	0.0	3.8	12.5	30.0	22.5	31.3	3.8
	CC8	35	4.8	5.7	0.0	0.0	0.0	5.7	0.0	20.0	31.4	17.1	37.1	5.7	8.6
	CC64	28	3.9	0.0	0.0	0.0	0.0	0.0	0.0	3.6	3.6	46.4	35.7	7.1	7.1
	CC45	25	3.4	0.0	0.0	0.0	0.0	0.0	0.0	4.0	4.0	8.0	52.0	32.0	4.0
	CC13	24	3.3	10.5	0.0	0.0	0.0	0.0	0.0	0.0	8.3	29.2	58.3	0.0	4.2
	CC3	19	2.6	13.3	0.0	0.0	0.0	0.0	0.0	0.0	10.5	31.6	36.8	15.8	5.3
	ST12	15	2.1	27.3	0.0	13.3	0.0	0.0	0.0	26.7	60.0	20.0	20.0	0.0	0.0
	CC83	11	1.5	0.0	0.0	0.0	9.1	0.0	0.0	0.0	9.1	18.2	9.1	63.6	0.0
C. malonaticus (136)	CC7	58	42.6	3.4	0.0	0.0	0.0	22.4	15.5	15.5	56.9	12.1	25.9	0.0	5.2

^a^Number of strains for each species in PubMLST database. See Table 1.

^b^Percentage of strains in each clonal group.

^c^No clinical presentation for infant isolate known.

^d^Necrotizing enterocolitis.

^e^Clinical, unknown presentation and host information.

The index of association (I_A_) is a measure of the linkage of a population. The I_A_ values for the genus *Cronobacter* was found to be significantly greater than zero (p < 0.001), indicating the presence of linkage disequilibrium or clonality [16, 17]. Clonal complexes are the STs which share 3 or more loci to the central ST. This recognises there are variants of each allele, for example single and double locus variant (SLV, DLV). A number of these large clonal complexes are especially significant with respect to strain clustering according to their isolation sources which will be considered in the next section.

The majority of *Cronobacter* infections, albeit less severe, are in the adult population [12]. An age profile of *Cronobacter* isolated using throat swabs from over 45,000 outpatients during the period 2005–2011 has been published [23]. The organism was isolated from every age group with a higher frequency from children less than 14 years of age. However the speciation and sequence type of these clinical isolates has not been published. As neonates are frequently fed reconstituted powdered infant formula (PIF), which is not a sterile product, this potential vector has been the focus of attention for reducing infection risk to neonates as the number of exposure routes is limited. It should be noted, however, that not all have been associated with reconstituted formula ingestion, and the organism has been isolated from the feeding tubes of neonates not receiving reconstituted powdered infant formula [24].

**Figure 2:**
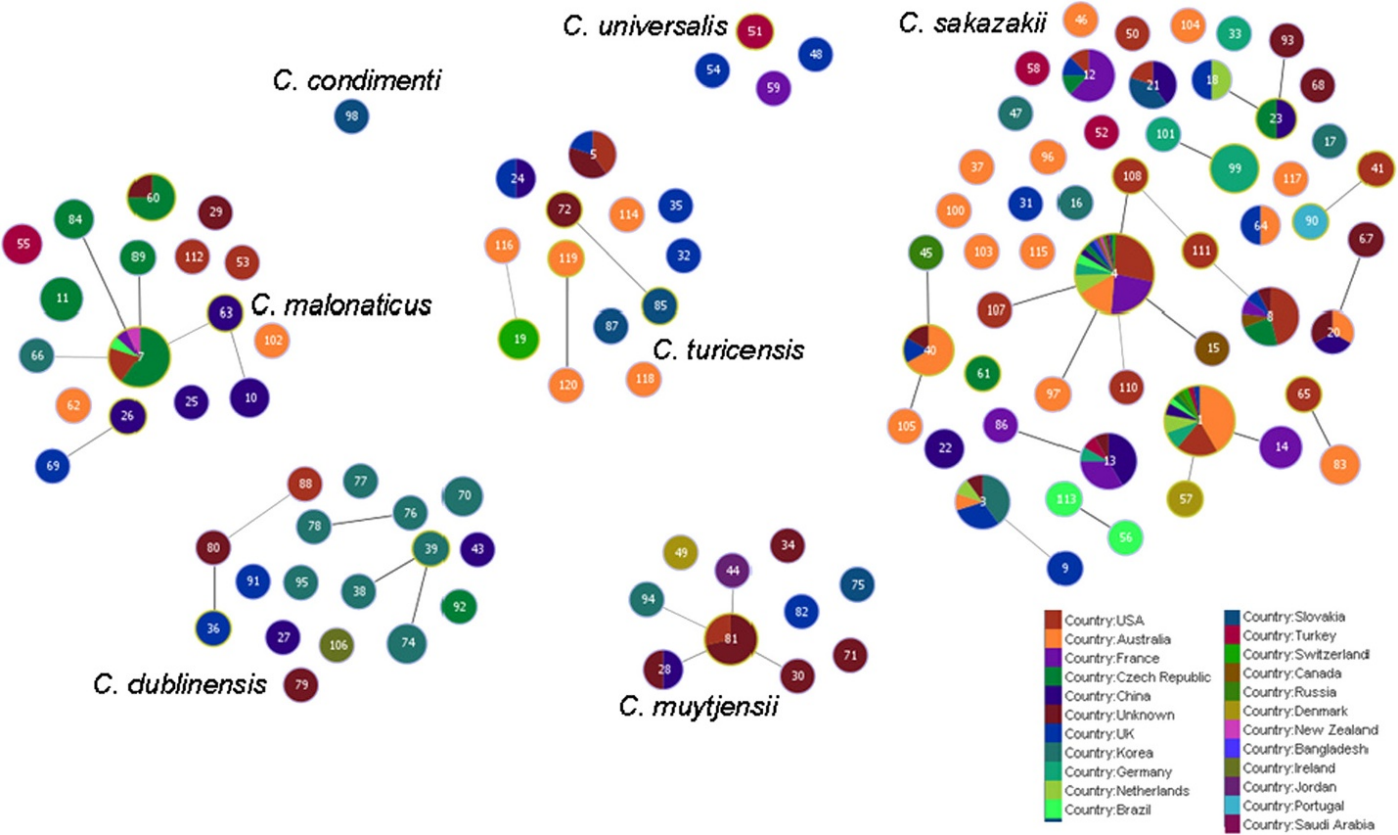
**Population snapshot of the** ***Cronobacter*** **MLST database generated using the GoeBURST algorithm, indicating the clonal complexes and the diversity of the sources of the strains.** The threshold for the output was set to triple locus variation. The dominant STs are represented by the circles with larger diameters. From Joseph & Forsythe, 2012.

GoeBURST analysis shows the formation of clonal complexes among the 298 identified STs for the *Cronobacter* genus (Figure 2), and an uneven distribution of clinical isolates across both the species and sequence types [17, 25, 26]. Some of these large clonal complexes are especially significant with respect to strain clustering according to their isolation sources. All sequence types can be analysed from the open access database which is updated regularly, however for convenience only selected ones are considered in detail below.

Clonal complex 4 (CC4) comprises single and double loci variants of *C. sakazakii* ST4. Currently these strains comprise 19.4% (n = 195) of the *Cronobacter* genus database. Forty-five percent (88/195) of these are clinical isolates and 24.6% (48/195) have been isolated from powdered infant formula; Table 2. This analysis demonstrates the importance of this clonal lineage with respect to *Cronobacter* spp. epidemiology as CC4 has been identified as a genetic signature for the *C. sakazakii* meningitic pathovar. Previously Joseph and Forsythe announced the strong association between neonatal meningitis cases and *C. sakazakii* ST4 using a retrospective study of only 41 clinical strains from 1953 to 2008, collected from 7 countries [27]. This association was confirmed by the analysis of 15 USA cases in 2011 [26]. These two earlier predictions of the association of *C. sakazakii* CC4 with meningitis are confirmed in Table 2 where, from over a hundred clinical isolates, this clonal lineage is prevalent and is recovered more frequently than other sequence types from infant infections especially cases of meningitis (20/23 cases). The reason for the association of one clonal complex in the genus with neonatal meningitis is unclear at present as no particular virulence traits have been determined in *C. sakazakii* CC4 compared to other sequence types [17].

It is notable that 24.6% (n = 195) of *C. sakazakii* CC4 isolates were from infant formula and also that 16.9% of isolates were from environmental sources such as milk powder and infant formula manufacturing plants; Table 2. This supports the previous individual reports of isolates from in Ireland and Switzerland [28, 29]. Sonbol et al. [30] reported that *C. sakazakii* CC4 accounted for 25% of *Cronobacter* strains isolated from the environment of 6 milk powder manufacturing plants in Australia and Germany, as well re-identified strains from an international survey in 1988 of *Cronobacter* in PIF. The finished genome sequence of a *C. sakazakii* CC4 strain (SP291) has been published, and was notable for its persistence in a PIF production facility in Ireland [29]. They also identified stress response and antimicrobial resistance genes in the genome. Furthermore, Muller et al. [28] used pulsed-field gel electrophoresis to match environmental isolates of *C. sakazakii* CC4 in a PIF manufacturing plant in Switzerland with those in the finished product. Therefore *C. sakazakii* CC4 may represent a particularly persistent clonal variant resulting in increased neonatal exposure. Whether the association of *C. sakazakii* CC4 with neonatal meningitis is due to greater neonatal exposure as a result of environmental fitness (i.e. desiccation persistence) or particular virulence capabilities is uncertain [17, 27]. Therefore an improved understanding of *C. sakazakii* CC4 strains is warranted to understand its prevalence both in PIF manufacturing plants and severe neonatal infections. New genomic profiling of *C. sakazakii* CC4 strains is presented in the last section.

*C. sakazakii* clonal complex 1 (CC1) is the next prominent ST consisting of 80 strains (11% of *C. sakazakii* strains in the database) isolated from around the world over a period of more than 25 years; Tables 1 and 2. These have been isolated from PIF and from milk powder processing factories [28, 30]. There are proportionately fewer (12.5%) CC1 clinical isolates compared to CC4 (45.1%), and only three (3.8%) of these strains are associated with meningitis [10]; Table 2. *C. sakazakii* CC1 strains were isolated from infant formula in 1994 used during a neonatal intensive care unit *Cronobacter* spp. outbreak in France [31]. Those strains did not match isolates from the infected neonates according to pulsed-field gel electrophoresis, and which were later shown to be *C. sakazakii* CC4 Also linked to this clonal complex is ST57 (DLV to ST1), which is the profile of a PIF isolate from Denmark in 1988. There are no known isolates of CC1 from adult patients which may indicate an adaptation to host by other clonal lineages.

Forty percent of *C. malonaticus* strains (n = 136) recorded in the database are in clonal complex 7. Strains in this complex have been isolated over the past 30 years. Within this complex 57% (33/58) of strains are clinical in origin and primarily from children and adults; Table 2. Only one reported fatal neonatal meningitis case has been attributed to *C. malonaticus*, though the vehicle of infection is uncertain and not necessarily linked to the consumption of infant formula [26]. The database only contains seven *C. malonaticus* CC7 isolates from PIF indicating a low incidence of this complex, and there are no isolates from infant formula or milk powder manufacturing plants.

Table 2 also shows for the first time that *C. sakazakii* ST12 has been associated with cases of necrotizing enterocolitis (13% of strains) and not neonatal meningitis or septicaemia. Although there are isolates in the database from infant formula, unlike CC4 there have been none from milk powder or infant formula manufacturing plants. The remaining clonal groups in Table 2 are less clinically relevant, and are more food and environmental isolates.

The online *Cronobacter* MLST database has therefore enabled the open recording of sources of *Cronobacter* isolates and can be interrogated by researchers, industry and regulatory authorities. The recording of the isolates is standardized and therefore facilitates an international contribution to collating information.

### Ecology of *Cronobacter*spp

Table 2 shows all seven *Cronobacter* species have been isolated from food and food ingredients and these comprise 14.2% of the total profiled isolates. These are primarily from plant material including herbs and spices [22, 32]. This is reflected in common physiological features which have been found in the genome [32–34]: yellow pigmentation for oxygen radical protection, capsule formation (adherence to plant surfaces) and efflux pumps (resistance to essential oils). Since plants are common food ingredients, these *Cronobacter* strains have been isolated from salads, cake mixes, packet soup, flavoured teas as well as powdered infant formula and weaning foods [35]. Table 2 shows that *C. sakazakii* CC4 strains are not so frequently isolated from food and food ingredients as *C. malonaticus* CC7; 8.2% compared to 25.9% of isolates. This may reflect the ecological diversity of the various clonal types and adaptation to niche. Another source for *Cronobacter* given in the database is water where 14/38 strains are *C. dublinensis*, and 13/38 are *C. sakazakii*, with no *C. sakazakii* ST4 strains. Despite one reported serious infection which can be attributed to reconsititution with water containing *Cronobacter*, prior to this analysis, water as a source of the bacterium has not received much attention [26].

### Taxonomy of the *Cronobacter*genus

Control of the organism requires a clear definition of the organism to distinguish it from closely related organisms which may be co-recovered. Accurate bacterial taxonomy is therefore essential for regulatory control because the detection methods must be based on a thorough understanding of the diversity of the target organism. A number of early *Cronobacter* detection methods were based on small numbers of poorly characterized, even misidentified, strains and therefore are not necessarily reliable for their stated purpose.

The application of NGS enabled an improved understanding of the taxonomy of the *Cronobacter* genus. The *Cronobacter* genus belongs to the bacterial class gammaproteobacteria, and is within the family *Enterobacteriaceae* with the nearest relatives being the newly described genus *Kosakonia* as well as the more familiar genera *Citrobacter* and *Pantoea.* Members of the latter include *Citrobacter koseri* which is notable as it is associated with invasive neonatal meningitis with clinical presentations of brain abscess formation. These symptoms are similar to those of *Cronobacter* spp. meningitis, and differ from those of the neonatal meningitis pathovar *E. coli* K1. Also members of the *Pantoea* spp. are primarily plant-borne and frequently are yellow pigmented as per the majority of *Cronobacter* isolates. *Enterobacter hormaechei* and *E. ludwiggii* isolates have been misidentified as *Cronobacter* which has led to some confusion in the literature [32].

Before the first (2004) FAO-WHO expert meeting yellow-pigmented *Enterobacter cloacae*-like bacterial strains were called *Enterobacter sakazakii.* Following further analysis, the *Cronobacter* genus was initially proposed for all previous *E. sakazakii* strains. The *Cronobacter* species were differentiated by Iversen et al. [15] according to 16 *E. sakazakii* biotypes; *C. sakazakii* (biotypes 1–5, 7, 8, 9, 11, 13 and 14), *C. turicensis* (biotypes 16, 16a and 16b), *C. muytjensii* (biotype 15), and *C. dublinensis* (biotypes 6, 10 and 12). This was quickly revised [36] with the addition of *C. malonaticus* (biotypes 5, 9 and 14). This latter species had originally been described as a sub-species of *C. sakazakii* by Iversen et al. [15] who could not distinguish *C. sakazakii* and *C. malonaticus* using 16S rDNA sequence analysis. Distinguishing between the two species had been problematic primarily for two reasons. Firstly, the use of biotype profiles to designate the *Cronobacter* species was not totally robust as a few of the biotype index strains were themselves assigned the wrong species [16, 37]. Secondly, there are seven copies of the rDNA gene in *Cronobacter* and intrageneric differences can lead to uncertain and inconsistent base calls with subsequent errors in GenBank entries.

In contrast, Joseph et al. [17] used strains selected by the seven loci multilocus sequence analysis (MLSA; 3036 bp concatenated sequence length) as representatives across the genus and therefore overcame the preconceived grouping of strains based on phenotyping. These studies led to the naming of two further *Cronobacter* species; *C. universalis* and *C. condimenti* [38] which as shown in Figure 1 are in the *Cronobacter* genus. Such recent changes in *Cronobacter* taxonomy are easily adapted using the sequence-based MLST scheme, whereas a number of currently approved methods including PCR probes require re-evaluation. In 2013, Brady et al. [39] used MLSA with only four loci (*gyrB, rpoB, infB* and *atpD*) for the re-evaluation of the taxonomic status of *Enterobacter helveticus*, *E. pulveris* and *E. turicensis*. Consequently these were renamed *C. helveticus*, *C. pulveris* and *C. zurichensis*, respectively. This particular re-evaluation was debatable due to the limited number of loci used which is below the normal permitted number of five. This taxonomic change also cause problems with the international control of *Cronobacter* spp. in powdered infant formula as many detection methods were designed using these *Enterobacter* species as negative controls [40, 41]. Soon after Stephan and colleagues proposed the latter three species should form two new genera named *Franconibacter* and *Siccibacter* [42]. Figure 1, using 7-loci MLSA confirms this taxonomic revision they do not cluster with the seven *Cronobacter* species.

The issue of whether all *Cronobacter* species are of clinical significance and need to be controlled in powdered infant formula, and other foods has not been considered by regulatory authorities. The lack of epidemiological evidence of infection from all species cannot be substantiated due to the frequent mis-identification of *Cronobacte*r strains following the routine use of phenotyping for identification. However as shown in Table 2, only a few *C. sakazakii* and *C. malonaticus* clonal groups have been isolated from neonatal clinical cases. The other species are primarily environmental commensals and are probably of little clinical significance. Whether infections have been unreported due to misidentification is highly probable as commercially available phenotyping kits (approved in ISO and FDA detections method protocols) still use the name *E. sakazakii*, and these also identify many true *Cronobacter* isolates as *Enterobacter cloacae*, *Pantoea* spp. or *Erwinia* spp.

Many MLST databases are now implemented using the Bacterial Isolate Genome Sequence Database (BIGSdb) platform [1, 43]. This enables a range of MLST-like schemes to be described and applied to whole genome sequenced organisms, in addition to the conventional laboratory-derived seven loci. Hence hierarchical classifications, either pre-defined in the database or user-defined, can be applied with progressively greater resolution as the number of loci analysed increases. In addition to the seven loci MLST scheme, as part of the *Cronobacter* site, a specific open-access repository for all *Cronobacter* genomes sequenced to date has been established. This enables the scalable analysis of *Cronobacter* genomes, representing all recognised species, for genes of interest and easily accommodates any changes in taxonomy. The *Cronobacter* seven loci MLST scheme has recently been extended online to include *ompA* and *rpoB* sequences such that these loci can add to users’ taxonomic evaluations (Tax-MLST).

**Figure 3:**
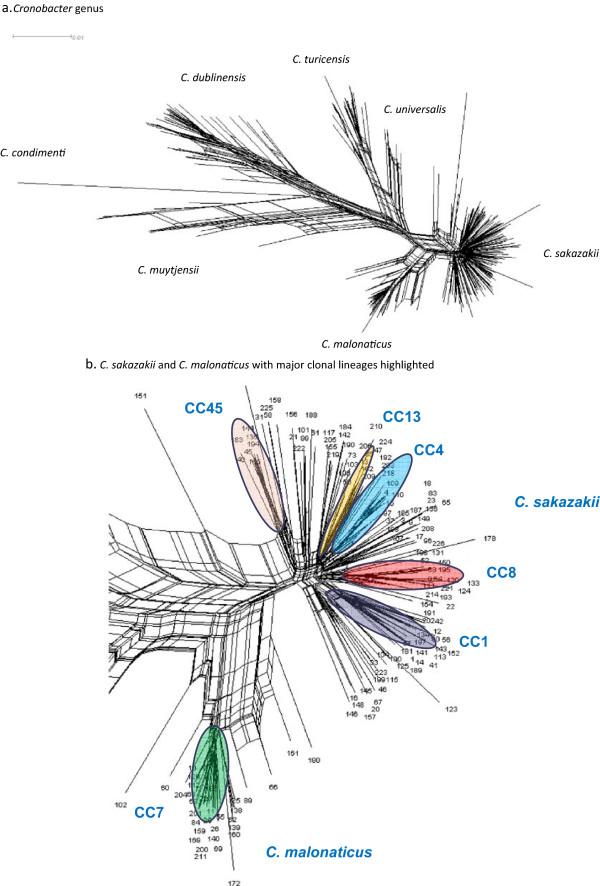
**7-loci Splits Network of 298 sequence types from the** ***Cronobacter*** **genus. (a)** Analyis of the whole Cronobacter genus **(b)** Analysis of C. sakazakii and C. malonaticus.

The separation of species is not always easy to define, and a large number of recombination events can occur within species, resulting in multiple plausible but only partially consistent taxonomic trees. Consequently NeighborNet analysis using Splitstree [44] has been applied to the genome sequence data of *Cronobacter.* Whereas the separation of *C. sakazakii* and *C. malonaticus* using 16S rRNA gene sequencing was problematic, the two species are clearly separated using the seven loci MLST (Figure 3 a,b). The figure also shows the considerable genetic diversity of *C. dublinensis* and *C. muytjensii* which has not been reported before. This reflects that there is a relative large number of sequence types for these two species. As given in Table 1, although even combined they are only represent 9.4% of the recorded isolates, the 94 strains are composed of 58 sequence types. These species are primarily isolated from environmental sites and are of less clinical relevance. In contrast, *C. sakazakii* and *C. malonaticus* form distinct clusters, within which the *C. sakazakii* CC4 and *C. malonaticus* CC7 clonal complexes can be distinguished (Figure 3b).

**Figure 4:**
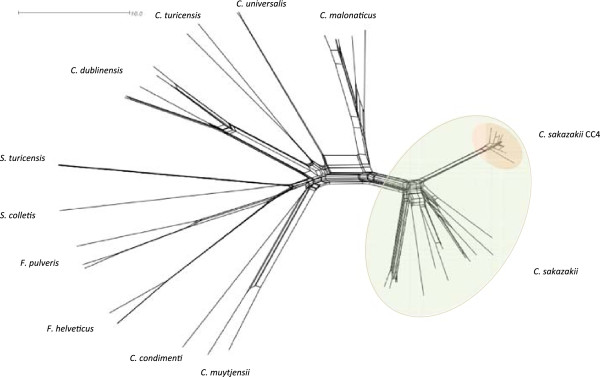
**rMLST analysis of genomes across the** ***Cronobacter*** **,** ***Franconibacter*** **and** ***Siccibacter*** **genera.**

Further multi-allelic analysis using genes coding for the ribosomal proteins (rMLST, 51-loci) has been used to integrate microbial genealogy and typing [2]. Expanding the multi-allelic analysis of *Cronobacter* spp. to rMLST shows the *C. sakazakii* CC4 clonal lineage as more pronounced and separate from the remaining *C. sakazakii* (Figure 4). This is based on 51 of the 53 ribosomal protein sequences due to the absence of two genes (*rpsA* and *rpsQ*) in some strains in the sample group. This reveals the *C. sakazakii* ST4 clonal lineage as defined by just seven loci is robust, and is also found using 51 other housekeeping genes. The relevance being this sequence type is strongly associated with neonatal meningitis infections and has not been genotyped to this level before.

**Figure 5:**
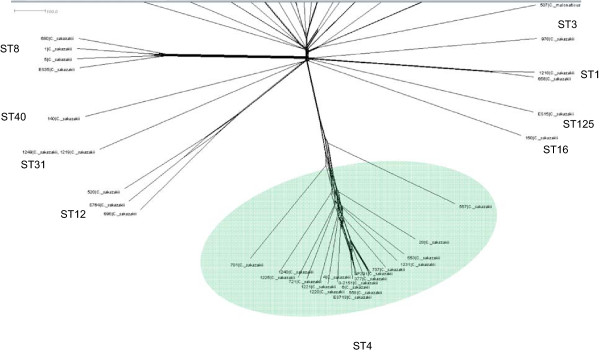
**COG-cgMLST analysis of** ***Cronobacter sakazakii*** **genomes. 7-loci MLST sequence types highlighted.**

Furthermore, genes tagged as belonging to Clusters of Orthologous Genes (COG) can be used to generate COG-cgMLST with 1865 loci [45]. Further expansion of the genomic analysis using COG-defined genes of *C. sakazakii* ES15 as the reference genome generated 1865 loci which could be compared across the genus or selected species or strains. COG loci are identified by their being tagged as belonging to COGs in *C. sakazakii* ES15 (excluding loci named in other analysis schemes). The subsequent figure also highlights the *C. sakazakii* clonal lineage CC4; Figure 5. COG-cgMLST analysis can also be used to generate a profile of potential biochemical traits which may be of use for differentiating strains and species using phenotyping. This is particularly useful as previously the choice of biochemical tests to try has been arbitrary. In addition the scheme can be used to study a range of physiological traits. Table 3 summarises the presence and variation in genes associated with cell division [46]. The core FtsLBQ cell division complex and FtsE/FtsX ABC transporter components are found in all *Cronobacter, Franconibacter* and *Siccibacter* genomes, whereas *ftsK* is only present in *C. sakazakii, C. malonaticus, C. universalis* and *C. turicensis*. This variation in cell division proteins may be linked to environmental stress tolerance. Therefore the PubMLST scheme not only enables molecular typing of *Cronobacter* isolates, but also readily accessible investigative profiling of strains.

**Table 3:** **Presence of cell division genes across the** ***Cronobacter*** **genus and closely related genera** ***Franconibacter*** **and** ***Siccibacter***

Gene	Function	***C. sakazakii***(33) ^a^	***C. malonaticus***(5)	***C. universalis***(2)	***C. turicensis***(2)	***C. muytjensii***(2)	***C. dublinensis***(5)	***C. condimenti***(1)	***F. helveticus***(2)	***F. pulveris***(3)	***S. turicensis***(3)	***Candidatus***S. colletis (1)
*ftsL*	FtsLBQ cell division complex	33	5	2	2	2	5	1	2	3	3	1
*ftsB*	FtsLBQ cell division complex	33	5	2	2	2	5	1	2	3	3	1
*ftsQ*	FtsLBQ cell division complex	33	5	2	2	2	5	1	2	3	3	1
*ftsY*	Signal recognition particle protein translocation system	32	5	2	2	2	5	0	2	3	3	1
*ftsE*	FtsE/FtsX ABC transporter	33	5	2	2	2	5	1	2	3	3	1
*ftsX1*	FtsE/FtsX ABC transporter	33	5	2	2	2	5	1	2	3	3	1
*ftsX2*	FtsE/FtsX ABC transporter	28	5	2	2	1	4	1	2	3	3	1
*ftsI*	Penicillin-binding protein 3	33	5	2	2	2	5	1	2	3	3	1
*ftsW*	Lipid II flippase	33	5	2	2	2	5	1	2	3	3	1
*ftsA*	Essential cell division protein	33	5	2	2	2	5	1	2	3	3	1
*ftsZ*	Essential cell division protein	33	5	2	2	2	5	1	2	3	3	1
*zipA*	Essential cell division protein	33	5	2	2	2	5	1	0	0	0	0
*ftsN*	Uncertain	33	5	2	2	2	5	1	0	3	0	0
*ftsK*	DNA translocase	29	5	1	2	0	0	0	0	0	0	0

^a^Number of genomes analysed.

### *Cronobacter*spp. virulence traits

*Cronobacter* can invade human intestinal cells, replicate in macrophages, and invade the blood brain barrier [47, 48]. The route of infection is probably through attachment and invasion of the intestinal cells. Whole genome sequencing has revealed a large number of plausible virulence factors, though many require further laboratory studies for confirmation [32–34]. Additionally the genomes of 107 *Cronobacter* strains, including 50 *C. sakazakii* ST4, are available for BLAST analysis and comparative genomic analysis via the PubMLST database.

Ten fimbriae clusters have been identified in the genomes of *Cronobacter* species [34]. Many fimbriae clusters are common to all species, though there are two interesting exceptions. *C. sakazakii* is the only *Cronobacter* species encoding for β-fimbriae, whereas the genomes of the other species encode for curli fimbriae. This may reflect evolution to the host ecosystem. A number of iron assimilation mechanisms have been found in *Cronobacter* species [49] which might enable the organism to utilize iron from breast milk and formula. Five putative Type VI secretion system (T6SS) clusters have been identified in *Cronobacter* spp. genomes. These may be involved in adherence, cytotoxicity, host-cell invasion, growth inside macrophages and survival within the host. It has been proposed that the outer membrane proteins ompA and ompX have roles in *Cronobacter* penetrating the blood brain barrier [50]. The mechanism(s) leading to the destruction of the brain cells is unknown and could in part be a host response. The organism also encodes for a number of haemolysins [34]. The organism has several plasmid borne features of interest including encoding for an outer membrane protease (*Cronobacter* plasminogen activator) that has significant identity to proteins belong to the Pla subfamily of omptins. Members of this subfamily of proteins degrade a number of serum proteins, including circulating complement, providing protection from the complement-dependent serum killing [51]. Having 107 genomes representing the *Cronobacter* genus in a curated, open-access database will facilitate the further analysis of these and other virulence traits. For example, using the BLAST facility shows the *cpa* gene is present in *C. sakazakii* strains (n = 72 genomes) but not *C. malonaticus* (n = 14 genomes) which may partially explain the variation in host susceptibility.

### Other *Cronobacter*characteristics of interest

Comparative genomic studies revealed that *C. sakazakii* possessed the *nanATKR* gene cluster which encodes for the utilization of exogenous sialic acid, and this may have clinical significance [34, 52]. The ability to utilise sialic acid as a carbon source could be a major evolutionary host-adaptation since the compound is found in breast milk, mucin and gangliosides. Sialic acid is also an ingredient in powdered infant formula due to its association with brain development. Laboratory studies designed to confirm the sialic acid utilization was active in *C. sakazakii* and also showed the species was able to grow on the ganglioside GM1 as a sole carbon source [52]. In contrast, unlike other *Cronobacter* species, *C. sakazakii* is unable to use malonic acid. Genomic analysis shows the malonate decarboxylase genes are next to an auxin efflux carrier which may function in malonate uptake. Malonic acid is an organic acid which is found in plant tissues and reflects the plant-association of the *Cronobacter* genus*.* The adaptation of *C. sakazakii* to a new ecosystem, with the subsequent loss of malonate utilization and gain in sialic acid utilization might contribute to this clinical significance. *Cronobacter* genomes also show the presence of a number of heavy metal resistance traits (copper, silver, zinc, tellurite) and capsule formation which might enable it to resist disinfectants in food production environments.

Hence the *Cronobacter* PubMLST database enabled researchers to study the detailed ecology, taxonomy and virulence of the organism. A timeline of key *Cronobacter* events are given in Additional file 1: Box 3, and list the various contributions of the *Cronobacter* PubMLST database.

## Conclusions

This paper demonstrates the application of NGS to *Cronobacter,* an emergent bacterial pathogen which had been poorly defined as a single species *Enterobacter sakazakii*. The advantages of a centralised multilocus sequence typing (MLST) database (i.e., http://pubmlst.org/cronobacter/) for genotyping the organism and recognising clinically relevant clonal lineages has been demonstrated using just seven loci. These lineages have been confirmed by 51-loci rMLST and even whole genome allelic profiling to the clone level. In addition, the three FAO-WHO key requests have been addressed by implementing this database using the BIGSdb platform, enabling the inclusion of 107 complete genomes within the 1007 recorded isolates. This database has also enabled the retrospective analysis of historic cases and outbreaks following re-identification of those strains and hence minimized the loss in information following taxonomic re-evaluations.

The multilocus sequence typing (MLST) and analysis (MLSA) approaches have:

- Revealed the diversity of the genus
- Contributed to the recognition of new species
- Shown the relatedness of the species,
- Shown the evolutionary decent of the genus
- Revealed the majority of neonatal meningitis cases are being attributable to one clonal lineage, *C. sakazakii* ST4 (CC4)
- Form the basis for future research regarding strain selection for investigating *Cronobacter* virulence and environmental fitness.

The *Cronobacter* PubMLST database offers a central, open access, reliable sequence-based repository. It has the capacity to create new analysis schemes ‘on the fly’ and integrate metadata (source, geographic distribution, clinical presentation). The database is able to adapt to changes in taxonomy and offers expandable analysis through BIGSdb implementation.

By producing a phylogenetic network for increasingly large fractions of the genome, rMLST and COG-cgMLST facilitate the identification of sequence types and species, while illustrating the ambiguities inherent in phylogenetic reconstruction across a genus. The application of Next Generation Sequencing has not only met the objectives of the 2004 FAO-WHO tasks, but continues to exceed them as the number of sequenced genomes has progressed.

## Methods

### Source DNA sequences

DNA sequences collated at http://pubmlst.org/cronobacter/ were investigated and Table 1 gives a summary of these strains. *In silico* analyses were carried out using options on the *Cronobacter* PubMLST portal accessible at: http://pubmlst.org/perl/bigsdb/bigsdb.pl?db=pubmlst_cronobacter_isolates.

### Seven loci MLST analysis

Concatenated sequences of seven loci from 298 sequence types were downloaded in FASTA format using the Export/Sequences option. These sequences were aligned in MEGA version 6.05 using the ClustalW algorithm. The final alignment spanned 3036 bp and was analysed using the default pipeline in SplitsTree4 (UncorrectedP to calculate distances and NeighborNet to build the network).

### rMLST analysis

A distance matrix for 107 *Cronobacter* genomes with available whole-genome sequences (and representing the whole genus [2]) was calculated with the Analysis/Genome Comparator option using default settings (with tagged allele designations used if available and completely excluding truncated loci). The matrix was exported in nexus format and analysed using the default pipeline in SplitsTree 4 (as above).

### COG-cgMLST scheme set-up and analysis

COG-cgMLST uses genes annotated as belonging to Clusters of Orthologous Groups [45] to define 1865 loci. The locus list, including reference sequences from *Cronobacter sakazakii* ES15, was assembled using an in-house script which identified CDSs in Genbank accession CP003312.1 in which the < note > field included a COG identifier. Sequences from these tagged loci were extracted and uploaded to the *Cronobacter* sequence definitions database excluding loci already named therein. Further scripts were used to establish COG-cgMLST locus scheme initially containing 1866 loci. The scheme was subsequently edited to exclude the locus *ppsA* (identical to *pps* in MLST) yielding 1865 loci.

With the scheme established, analysis was carried out as above with the use of the Analysis/Genome Comparator option and the default pipeline in SplitsTree4 (as above). Note that the Genome Comparator option with COG-cgMLST will analyse samples directly against the reference-defined loci and not with reference to COG IDs in query genomes.

## Electronic supplementary material

Additional file 1: **Box 1.** FAO-WHO executive summary (2004); see footnote. **Box 2. Box 3.** Summary timeline of Cronobacter recognition, control and molecular profiling [53]. (DOC 30 KB)

## Abbreviations

BIGSdb: Bacterial Isolate Genome Sequence Database
CC: Clonal complex
cgMLST: Core genome multilocus sequence typing
COG: Clusters of Orthologous Groups
COG-cgMLST: Clusters of Orthologous Groups-core genome multilocus sequence typing
FAO-WHO: Food and Agricultural Organization-World Health Organization
MLSA: Multilocus sequence analysis
MLST: Multilocus sequence typing
NGS: Next Generation Sequencing
NEC: Necrotizing enterocolitis
PIF: Powdered infant formula
rMLST: Ribosomal multilocus sequence typing
ST: Sequence types
Tax-MLST: Taxonomic multilocus sequence typing
wgMLST: Whole genome multilocus sequence typing.
