# Supplementary material for: Cronobacter, the emergent bacterial pathogen Enterobacter sakazakii comes of age; MLST and whole genome sequence analysis
Source: BMC Genomics. 2014 Dec 16;15(1):1121. doi: 10.1186/1471-2164-15-1121 (PMC4377842; doi:10.1186/1471-2164-15-1121)
Supplement: Supplementary file 1 — Additional file 1: Box 1. FAO-WHO executive summary (2004); see footnote. Box 2. Box 3. Summary timeline of Cronobacter recognition, control and molecular profiling [53]. (DOC 30 KB) [file 12864_2014_6836_MOESM1_ESM.doc]

Additional file 1

Box 1. FAO-WHO executive summary (2004); see footnote.

1. The use of internationally validated detection and molecular typing methods for *Cronobacter* spp. and other relevant microorganisms should be promoted.
2. Investigation and reporting of sources and vehicles, including powdered infant formula, of infection by *Cronobacter* spp. and other relevant microorganisms should be promoted.
3. Research should be promoted to gain a better understanding of the ecology, taxonomy, virulence and other characteristics of *Cronobacter* spp. and on ways to reduce its levels in reconstituted powdered infant formula.

Footnote: Please note the term ‘*Cronobacter* spp.’ has been used in place of ‘*E. sakazakii’* as used in the original text due to changes in taxonomy since the FAO-WHO report [10].

Box 2.

*Cronobacter* spp. formerly known as the single species *Enterobacter sakazakii*

Description: *Cronobacter* genus. Seven recognised species: *C. condimenti, C. dublinensis, C. malonaticus, C. muytjensii, C. sakazakii, C. turicensis, , C. universalis*, and *Candidatus* C. colletis*.* Gram-negative, frequently motile, facultatively aerobic rods, members of the *Enterobacteriaceae*.

*Clinical presentations:* Infections in all age groups, particularly neonates, infants <6 months in age, and the elderly. Life-threatening symptoms in primarily low birth weight neonates and infants <6 months (pre-weaning) in age include meningitis, necrotising enterocolitis, and respiratory infections. Symptoms in adults include bacteraemia, septicaemia, urosepsis, and wound infections.

*Source:* Infections in neonates associated with contaminated reconstituted powdered infant formula. Ubiquitous in nature, particularly plant material. Human carriage.

*Detection method:* Cultivation on chromogenic agar, followed by sequencing of *fusA* or *rpoB* gene for phylogenetic analysis.

Box 3. Summary timeline of *Cronobacter* recognition, control and molecular profiling.

Pre-2002 Various outbreaks in neonatal intensive care units, and sporadic cases reported [7,9]

2002 Outbreak at University of Tennessee investigated by CDC and FDA. Fatal case linked to use of powdered formula [8].

FDA *Cronobacter* (then *E. sakazakii*) detection method; Bacteriological Analytical Manual announced

2004 1^st^ FAO-WHO risk assessment meeting on microbiological safety of powdered infant formula [10]

Development of first chromogenic differential agar; DFI [21]

Outbreak in France, including two fatal meningitis cases. Four contaminated batches of PIF were linked to illnesses [10].

2006 2^nd^ FAO-WHO risk assessment meeting [11]

ISO detection method announced; ISO/TS 22964.

2008 3^rd^ FAO-WHO risk assessment meeting [12]

Revised Codex Alimentarius Commission guidelines for microbiological specifications for powdered infant formula released, to include *Cronobacter* as a named pathogen [19]

*Cronobacter* genus composed of 5 species defined [15]

2009 Open access seven loci multilocus sequence typing (MLST; 3036 bp) scheme established; [PubMLST.org/cronobacter](http://pubMLST.org/cronobacter)/ [16]

2010 First whole sequence for *C. sakazakii* published. Strain BAA-894 had been isolated during the University of Tennessee outbreak of 2002 [8,34]

2011 *C. sakazakii* sequence type 4 (clonal lineage 4) association with neonatal meningitis determined [28].

Two new *Cronobacte*r species recognised; *C. condimenti* and *C. universalis* [39].

2012 First pan-genome analysis of *Cronobacter* genus based on 14 genomes published [35].

Revised FDA method of detection; Bacteriological Analytical Manual [53]

2013 Three new *Cronobacter* species proposed: *C. helveticus*, *C. pulveris* and *C. zurichensis* [40]

[PubMLST.org/cronobacter](http://pubMLST.org/cronobacter)/ expanded to include:

1. Tax-MLST established, extension of MLST to include genotyping of *ompA* and *rpoB*
2. Searchable repository for all published *Cronobacter* spp. genomes established

2014 *C. helveticus*, *C. pulveris* and *C. zurichensis* taxonomy renamed *Franconibacter helveticus, F. pulveris* and *Siccibacter turicensis* [43]

[PubMLST.org/cronobacter](http://pubMLST.org/cronobacter)/ expanded to include:

1. rMLST (51 loci; 20kbp) scheme released
2. COG—cgMLST(1865 loci; 1.98 Mbp) scheme released
3. *Candidatus S*. colletis proposed, and genome published [41]
